# Exploring the Molecular Growth of Two Gigantic Half‐Closed Polyoxometalate Clusters {Mo_180_} and {Mo_130_Ce_6_}

**DOI:** 10.1002/anie.201702957

**Published:** 2017-06-01

**Authors:** Weimin Xuan, Robert Pow, De‐Liang Long, Leroy Cronin

**Affiliations:** ^1^ WestCHEM, School of Chemistry The University of Glasgow Glasgow G12 8QQ UK

**Keywords:** gigantic cluster, molecular growth, molybdenum blue, polyoxometalates, symmetry breaking

## Abstract

Understanding the process of the self‐assembly of gigantic polyoxometalates and their subsequent molecular growth, by the addition of capping moieties onto the oxo‐frameworks, is critical for the development of the designed assembly of complex high‐nuclearity cluster species, yet such processes remain far from being understood. Herein we describe the molecular growth from {Mo_150_} and {Mo_120_Ce_6_} to afford two half‐closed gigantic molybdenum blue clusters {Mo_180_} (**1**) and {Mo_130_Ce_6_} (**2**), respectively. Compound **1** features a hat‐shaped structure with the parent wheel‐shaped {Mo_150_} being capped by a {Mo_30_} unit on one side. Similarly, **2** exhibits an elliptical lanthanide‐doped wheel {Mo_120_Ce_6_} that is sealed by a {Mo_10_} unit on one side. Moreover, the observation of the parent uncapped {Mo_150_} and {Mo_120_Ce_6_} clusters as minor products during the synthesis of **1** and **2** strongly suggests that the molecular growth process can be initialized from {Mo_150_} and {Mo_120_Ce_6_} in solution, respectively.

Polyoxometalates (POMs) are a unique class of discrete metal‐oxo clusters with a diversity of structures and properties.^1^, ^2^, ^3^ The almost unlimited combination of anion templates and basic building blocks in solution results in an extensive library of POM clusters with a range of extraordinary structures. As such, particular attention has been paid to the grand goal of the rational design and synthesis of gigantic POMs clusters, building on prior structural and mechanistic data.^4^ This is because, in general, gigantic POMs clusters are constructed from a precisely ordered arrangement of building blocks which appear structurally to be fundamental to this class of compounds. For example, the molybdenum blue (MB) clusters such as {Mo_154_}, {Mo_176_}, and {Mo_368_}, are constructed from basic {Mo_8_}, {Mo_2_}, and {Mo_1_} building blocks,4a–4c and gigantic polyoxotungstates such as {W_224_Mn_40_} and {W_200_Co_8_} are generated via the connection of {W}_*X*_ (*X*=8–15) building blocks.4d,4e Nevertheless, it is rare to see reports on the use of gigantic preformed POMs as “big building blocks” to construct even higher nuclearity clusters, or to achieve high‐order assemblies.^5^ Two prominent examples are the molecular growth of {Mo_248_} from the smaller {Mo_176_}5a and in situ growth of metal‐oxo clusters such as {Cu_20_} and {Fe_16_} within the cavity of the ring shaped {P_8_W_48_}.5b,5c In principle, molecular growth based on gigantic POM clusters will not only result in novel clusters with high structural diversity, but also leads to the opportunity of introducing new building blocks/functionalities that cannot be discovered via traditional synthesis methods, opening up an alternative pathway to build functional POM‐based materials.^5^ Meanwhile, it is important to understand the way in which the additional fragments initialize the molecular growth process. This is particularly important to improve the understanding of the initial nucleation of POM clusters in solution, as well as the metal‐centered assemblies found in biological systems.^5^, ^6^ In this context, the investigation of clusters that can be grown/assembled by molecular growth processes is warranted.

Within the MB family of gigantic isopolyoxomolybdates clusters the {Mo_2_} units are relatively reactive and can be easily coordinated by carboxylic acids, or replaced by electrophiles, such as lanthanides, to afford amino acid‐functionalized^7^,^8^ or lanthanide‐doped^7^,^9^ MB species. More importantly, we hypothesized that the inward terminal O atoms on the {Mo_2_} units could provide interactive sites to support further growth from the inner surface, as evidenced by the molecular growth from {Mo_176_} to {Mo_248_}.5a Inspired by this, we set out to explore the molecular growth using a smaller MB, such as {Mo_154_}, and lanthanide‐doped MB as the parent scaffolds, to see how different sizes and curvatures of the pristine rings may affect the resulting clusters. Herein, we report two novel MB clusters {Mo_180_} (**1**) and {Mo_130_Ce_6_} (**2**) constructed via the molecular growth from {Mo_150_} and {Mo_120_Ce_6_}, respectively. Compounds **1** and **2** exhibit unprecedented half‐closed structural motifs arising from the asymmetric growth of cap‐like {Mo_30_} and {Mo_10_} units present on just one side of {Mo_150_} and {Mo_120_Ce_6_}, respectively (Scheme 1).

**Scheme 1:**
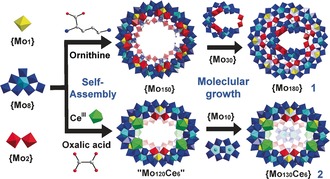
Schematic representation of molecular growth from {Mo_150_} to **1** and from proposed intermediate “Mo_120_Ce_6_” to **2**. {Mo_1_} yellow polyhedron, {Mo_2_} red polyhedron, {Mo_8_} blue polyhedron with central pentagonal unit in cyan polyhedron, Ce^III^ green polyhedron, C gray ball, N blue ball.

Compound **1** was prepared by reducing an acidified solution of Na_2_MoO_4_ and l‐ornithine in H_2_O while **2** was obtained from reduction of an acidified suspension of cerium polymolybdate in H_2_O. l‐ornithine was introduced as cationic structure‐directed agent during the self‐assembly which not only balances the overall charge of **1** but, also facilitates the further growth of capped fragment by adjusting the orientation and conformation of the {Mo_2_} units. All the compounds were characterized crystallographically and the formula assignments are fully supported by using an extensive array of analytical techniques (see Supporting Information). Compounds **1**–**2** can be formulated as [Eqs. (1), (2)]:

(1) $$\mathrm{Na}{}_{4}\left(C{}_{5}H{}_{11}N{}_{2}O{}_{2}\right){}_{2}\left\{H{}_{18}\mathrm{Mo}{}_{180}O{}_{536}\left(H{}_{2}O\right){}_{78}\left(C{}_{5}H{}_{10}N{}_{2}O{}_{2}\right){}_{7}\right\}\cdot 250\;H{}_{2}O\;\;\;\equiv \;\mathrm{Na}{}_{4}\left(C{}_{5}H{}_{11}N{}_{2}O{}_{2}\right){}_{2}\left\{1\;a\right\}\cdot 250\;H{}_{2}O$$

(2) $$\mathrm{Ce}{}_{0.5}\left[\mathrm{Ce}{}_{6}H{}_{16.5}\mathrm{Mo}{}_{130}O{}_{396}\left(H{}_{2}O\right){}_{84}\right]\cdot 180\;H{}_{2}O\;\equiv \;\mathrm{Ce}{}_{0.5}\left\{2\;a\right\}\cdot 180\;H{}_{2}O$$

The single‐crystal X‐ray structural analysis reveals that **1** crystalizes in the space group *P*−1 and not only has a ring topology, but has a another cluster grafted to the rim of the ring giving a hat‐like {Mo_180_} (**1 a**), composed of 17 {Mo_8_} units, 15 {Mo_2_} units, and 14 {Mo_1_} units (Figure 1 a,b). Compound **1 a** could be divided into two parts, that is, the brim of hat, {Mo_150_}, and the cap, {Mo_30_}. The {Mo_150_} adopts the same framework of archetypal {Mo_154_} discovered by Müller et al.^4^ that is constructed from 14 sets of {Mo_11_} units but with two {Mo_2_} sites with defects on the rim of the wheel (Figure 1 c). In total, there are five {Mo_2_} units on the lower rim and seven {Mo_2_} units on upper rim of {Mo_150_}. The {Mo_30_} cap is situated on top of {Mo_150_} and is connected to the main ring via 7‐based {Mo_2_} units. The {Mo_30_} is built from three {Mo_8_} units which are linked with two edge‐sharing {Mo_2_} units and one corner‐sharing {Mo_2_} unit (Figure 1 d). It should be noted that edge‐sharing {Mo_2_} units are also the basic building blocks for the spherical inorganic Fullerene Keplerate structure, {Mo_132_}, and have never before been observed in the structure of MB wheels.^10^ In addition, seven l‐ornithine ligands are found to be grafted onto the seven {Mo_2_} units with the side chain buried in the pitch of {Mo_150_}. Among them, two pairs of l‐ornithine are arranged in tail‐to‐tail mode with the terminal amino groups pointing to each other while another pair of l‐ornithine ligands adopt a head‐to‐head arrangement (Figure S4).

**Figure 1:**
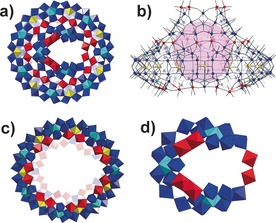
a) Top view of the molecular structure of **1 a** in polyhedron representation. {Mo_1_} yellow polyhedron, {Mo_2_} red polyhedron, {Mo_8_} blue polyhedron with central pentagonal unit in cyan polyhedron. l‐ornithine is omitted for clarity. b) Side view of hat‐like **1 a** in ball and stick mode. Mo color code same as in (a); O gray. The inserted purple ball is to highlight void space within **1 a**. c) and d) Polyhedron representation of {Mo_150_} (c) and {Mo_30_} (d).

The geometry and shape of **1 a** resembles that of half of a {Mo_368_} cluster.4c To make a better comparison, we have simplified both the structures and placed an emphasis on pentagonal {Mo_6_} units as shown in Figure 2. For example, in Figure 2 e, the 20 pentagons found in half of the {Mo_368_} cluster are shown tiled in three layers (A–C) with an 8‐8‐4 arrangement. The 8 pentagons in layer B stack between 8 pentagons in layer A and each pentagon in layer C further pack between 4 pairs of pentagons in layer B. Therefore, the general rule is that pentagons in three layers are connected in a ratio of 2:2:1 (Figure 2 b,e). Applying this rule to **1 a**, the ideal arrangement of pentagons in the three layers should be 7‐7‐3.5. In consideration of steric hindrance and spatial constraints, only three pentagons are built in layer C and each of them are located between two pentagons in layer B with the positon corresponding to a pentagon being left vacant, see the VS (vacant site) in Figure 2 a. Notably, this unoccupied or “defect” site superimposes on the position of the missing {Mo_2_} units on {Mo_150_}, indicating that both the brim and cap of **1 a** have adjusted their structures to dissatisfy the non‐defect‐based {Mo_154_} and the 3.5 pentagons to promote the formation of **1 a**. Similar to that of {Mo_368_}, symmetry breaking occurs between layer B and C in **1 a**, and the local symmetry of {Mo_150_} dramatically reduces from ideal *D*_7*d*_ to *C*_s_ in the cap. Accordingly, the positively curved interior surfaces of {Mo_150_} transform to negative curvatures in the cap. There are two domains of negative curvatures formed between {Mo_2_} units and {Mo_6_} pentagons in layer B and C (Figure 2 d and Figure S5). One domain contains two negative curvatures while another one comprises three negative curvatures (Figure S5).

**Figure 2:**
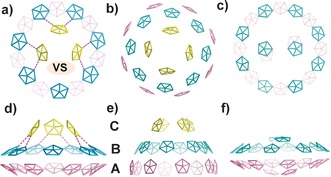
The simplified structure of **1 a** and half {Mo_368_} and {Mo_248_} in wire‐frame representation based on pentagonal {Mo_6_} units. Pentagons in different layers are colored: yellow (layer C), cyan (layer B), and rose (layer A). a) Top view of simplified **1 a**. b) Top view of simplified half {Mo_368_}. c) Top view of simplified {Mo_248_}. d) Side view of simplified **1 a**. Doted purple line is used to highlight the negative curvatures between layer B and layer C. e) Side view of simplified half {Mo_368_}. f) Side view of simplified {Mo_248_}. VS=vacant site normally occupied by a pentagonal unit.

In principle, the torus‐shaped {Mo_176_} and {Mo_154_} can mainly support the in‐plane growth of capping moieties along the direction of interior rim defined by {Mo_2_} units, as shown by the molecular growth from {Mo_176_} to {Mo_248_} which has a “flying saucer‐like” shape (Figure 2 c,f).4a,4b, 5a In view of {Mo_368_}, longitudinal growth is favored owing to the pumpkin‐shaped central parts (layer A+B, composed of 16 pentagons; Figure 2 e).4c In this respect, it is interesting to observe the molecular growth of the capping {Mo_30_} along the longitudinal axis of the {Mo_150_}. After a detailed analysis of the topological structure of **1 a**, we found that the {Mo_2_} units in **1 a** play a key role in directing the longitudinal growth. As shown in Figure 3 a and Figure 3 b, each Mo atom of the corner‐shared {Mo_2_} in both {Mo_248_} and {Mo_150_} uses two O atoms in the equatorial plane to link with adjacent {Mo_6_} pentagons (green lines in Figure 3) and the third O atom to support further growth of capping moiety (blue lines in Figure 3). The spatial arrangement of two Mo atoms in {Mo_2_} unit could be evaluated by the orientation of their equatorial planes. As indicated by the black lines plotted through the two apical O atoms, two Mo atoms of {Mo_2_} in {Mo_248_} are almost in parallel (Figure 3 a) while the counterpart in {Mo_150_} twist to each other and form a very acute angle between the two black lines in Figure 3 b. This is further supported by the dramatic decrease in distance between the two adjacent apical O atoms (doted purple lines in Figure 3) from 3.559 to 2.190 Å. The reason why {Mo_2_} units in {Mo_150_} take such a deformed configuration is due to the coordination of l‐ornithine. In contrast to the ligated water, the bidentate bridging carboxylate on l‐ornithine exerts a strong constraint on the Mo atoms in {Mo_2_} unit and thus pushes them to be much closer to each other (Figure 3 b). Indeed, this kind of {Mo_2_} unit is unique and can also be found in the cysteine and tyrosine functionalized {Mo_154_}.^8^ The different arrangements make the {Mo_2_} unit roughly stay in the same plane of two {Mo_6_} pentagons in the {Mo_248_}, but deviate greatly from the plane defined by pentagons in {Mo_150_} (Figure 3 a,b). Since the equatorial planes of two Mo atoms in {Mo_2_} change from approximately coplanar in {Mo_248_} to inclined to each other in {Mo_150_}, the potential growing directions (blue lines in Figure 3 a,b) shift from slightly pointing inwards to stretching outwards while both keep pointing upwards. In this way, the longitudinal growth of {Mo_30_} is achieved by connecting with {Mo_2_} units on {Mo_150_}.

**Figure 3:**
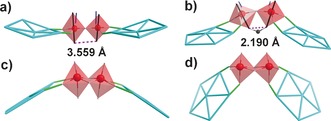
Top: The connectivity and spatial arrangement of corner‐shared {Mo_2_} units in {Mo_248_} (a) and {Mo_150_} (b). Bottom: the connectivity and spatial arrangement of edge‐shared {Mo_2_} units in {Mo_132_} (c) and the {Mo_30_} cap (d). {Mo_2_} units are presented in transparent red polyhedra with central Mo atoms as red balls. Two apical O atoms on adjacent Mo atoms are linked by black lines. The connection between {Mo_2_} and pentagons is highlighted as green lines and the connection between {Mo_2_} and capping moieties is indicated as blue lines. Pentagons are colored as cyan wires. The distance between adjacent apical O atoms on {Mo_2_} unit is represented by dotted purple line and the C atom on carboxylate of l‐ornithine as dark ball.

The edge‐shared {Mo_2_} units in {Mo_30_} also contribute to the longitudinal growth. As shown in Figure 3 c, each Mo atom of edge‐shared {Mo_2_} in {Mo_132_} use two O atoms from the equatorial plane to bind with the adjacent {Mo_6_} pentagons. Therefore, the slightly bent {Mo_2_} in {Mo_132_} mainly supports the horizontal growth along equatorial plane to afford Keplerate‐like sphere. In contrast, the {Mo_2_} in {Mo_30_} could make use of both the O atoms from equatorial plane and apical positions to do so (Figure 3 d). This type of connectivity means that the {Mo_2_} unit in the {Mo_30_} is prone to deviate from the positive camber defined by adjacent two pentagons, and thus facilitate the longitudinal growth instead of horizontal growth.

The successful synthesis of compound **1** promotes us to explore the potential of using smaller MB as scaffold to realize the molecular growth. To do this, we selected a lanthanide‐doped Mo Blue (LMB) as the candidate.^9^ In general, LMBs are prepared using strong electrophiles such as Ce^III^, Pr^III^, and Eu^III^ ions to replace {Mo_2_} units on the parent {Mo_154_}. The resulting LMB generally exhibits smaller dodecameric or decameric frameworks in comparison with tetradecameric {Mo_154_}. Since the Ln^III^ ions are significantly smaller than {Mo_2_} unit, the substitution of the {Mo_2_} units with Ln^III^ ions causes the contraction of the wheel, and consequently increases the curvature on the inner surface of the ring. Therefore, the incorporation of lanthanide ions not only adjusts the available number of {Mo_2_} units but also tunes the curvature and size of the MB. With this in mind, we envision that LMBs may provide a great chance to cap “species” differently from the iso‐POMs such as {Mo_150_} and {Mo_176_}.

After a systematic optimization of the synthetic conditions we were able to obtain the half‐closed compound **2**. Single‐crystal X‐ray structure analysis reveals that **2** crystalizes in space group *Pnma* and features an elliptical ring structure of {Mo_120_Ce_6_} capped by a {Mo_10_} unit on one side (Figure 4). The parent {Mo_120_Ce_6_} could be regarded as dodecamer that is composed of 12 {Mo_8_} units, 6 {Mo_2_} units, 12 {Mo_1_} units, and 6 Ce^III^ ions.9a, 9b The Six Ce^III^ ions are distributed on both upper and lower rim of the wheel in an unsymmetrical arrangement, i.e., 4 of them are located symmetrically on one side while another 2 stay on the same side of {Mo_10_} cap. The incorporation of 6 Ce^III^ ions on the inner surfaces of **2 a** greatly reduces the symmetry of the wheel to *C*_2*v*_ as compared with archetype {Mo_154_} (*D*_7*d*_ point group). From the crystal structure, the ideal composition of cap fragment should be {Mo_11_}, however, the central pentagonal Mo atoms could be disordered equally on two positions with the total occupancy of one, so the actual formula of the cap is {Mo_10_} (Figure 4 a). {Mo_10_} is composed of one {Mo_6_} pentagon and two corner‐shared {Mo_2_} units which are connected with four {Mo_2_} units on {Mo_120_Ce_6_} (Figure 4 a). Because the {Mo_2_} units in {Mo_120_Ce_6_} adopt the similar configuration as the one in {Mo_248_}, {Mo_10_} grows along the inner surface to afford the half‐closed **2 a** (Figure 4 a,b).

**Figure 4:**
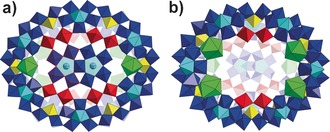
a) View of the molecular structure of **2 a** and {Mo_10_} cap. The disordered central pentagonal Mo atoms are highlighted as cyan balls and corner‐shared {Mo_2_} units in {Mo_10_} are bordered in red wires. b) View of **2 a** from the opposite side of {Mo_10_}. Color code of Mo is the same as Figure 1; Ce green polyhedron.

It appears that the Ce^III^ ions play a significant role in directing the formation of **2**. In general, Ln^III^ ions can adopt three different modes to link with adjacent {Mo_6_} pentagons in which they act as 2‐, 3‐, and 4‐connected rods, respectively (Figure S10).^9^ From the crystal structure, all the six Ce^III^ ions on **2 a** adopt a 4‐connected mode and stay localised at the two ends of the wheel creating an oval‐shaped {Mo_120_Ce_6_}, thus providing a suitable pocket on one side to support the growth of the elliptical {Mo_10_} moiety. It is therefore reasonable to speculate that if the connecting modes and the positions of Ln^III^ ions on the wheels could be manipulated in a controlled manner, then LMB with a variety of curvatures, shapes and sizes could be produced to not only enrich the structural library of LMB, but also provide more chances to use them as “functional building blocks”.

The synthesis of both **1** and **2** can only be achieved under quite specific conditions. More precisely, **1** must be synthesized in very dilute solution; increasing the concentration of the starting materials or altering the reducing agents will result in the formation of a series of l‐ornithine functionalized MBs, such as {Mo_150_}, {Mo_148_}, and {Mo_146_}, owing to the faster crystallization rate.^11^ In some cases, we also observed the coexistence of {Mo_150_} together with **1**. Based on this, we postulate the self‐assembly of **1** occurs as follows: Firstly, the basic building blocks of {Mo_8_}, {Mo_2_}, and {Mo_1_} aggregate into l‐ornithine functionalized {Mo_150_}. Owing to the dilute conditions, {Mo_150_} does not precipitate from solution, but instead reacts with the “active species” in solution to initialize molecular growth. In this situation, three {Mo_8_} units of the {Mo_30_} gradually build‐up on the {Mo_150_}, and the molecular growth is finally terminated by the two edge‐sharing {Mo_2_} units (Scheme 1). On the other hand, **2** is first found as side product during the synthesis of {Mo_120_Ce_6_}‐a which is an isomer of the parent {Mo_120_Ce_6_} within **2a** and isostructural to the reported {Mo_120_Pr_6_}.9b After addition of oxalic acid in the synthesis, **2** could be exclusively obtained in high yields (>30 %), although sometimes minor amounts of {Mo_120_Ce_6_}‐a could also be identified by microscope owing to its distinct crystal morphology and unit‐cell checking as well. We propose that oxalic acid serves as a transient anion template, or provides supramolecular interactions, such as hydrogen bonding, to promote the encapsulation of {Mo_10_} during the assembly process. In view of this we therefore speculate a potential pathway for the self‐assembly of **2**. In the absence of oxalic acid, {Mo_120_Ce_6_}‐a is produced as the major product, but with the aid of oxalic acid, {Mo_120_Ce_6_}, the parent MB framework of **2 a**, is formed in solution and then serves as parent scaffold to trap the {Mo_10_} cap on one side (Scheme 1).

In summary, we present two half‐closed MB clusters **1** and **2** derived from the asymmetric molecular growth from the parent clusters {Mo_150_} and {Mo_120_Ce_6_}, respectively. Compound **1** exhibits a unique hat‐shaped structure composed of the parent {Mo_150_} and a {Mo_30_} cap. In particular, several domains of negative curvatures are found to occur between the interfaces of the {Mo_30_} and {Mo_150_} owing to the mismatching symmetry between the units at the growth interface. Furthermore, the unique spatial arrangement of both corner‐sharing and edge‐sharing {Mo_2_} units in {Mo_180_} contributes to this curvature, and to the longitudinal growth of the {Mo_30_} cap. Compound **2** features a dodecameric lanthanide‐doped wheel {Mo_120_Ce_6_} that is sealed by a {Mo_10_} unit on one side. In contrast to **1**, {Mo_10_} grows along the rim of {Mo_120_Ce_6_} to give rise to a half‐closed wheel. The synthesis of **1** and **2** can only be achieved under quite specific conditions, and it is found that in some cases {Mo_150_} and {Mo_120_Ce_6_}‐a are observed as minor products during the self‐assembly of **1** and **2**, respectively. Based on this, we confirmed the molecular growth from {Mo_150_} and {Mo_120_Ce_6_} to **1** and **2**, respectively. In the future, we will further extend the concept of “molecular growth” to discover more novel gigantic POM clusters.

## Conflict of interest

The authors declare no conflict of interest.

## Supporting information

As a service to our authors and readers, this journal provides supporting information supplied by the authors. Such materials are peer reviewed and may be re‐organized for online delivery, but are not copy‐edited or typeset. Technical support issues arising from supporting information (other than missing files) should be addressed to the authors.

Supplementary
