# Supplementary material for: Exploring the Molecular Growth of Two Gigantic Half‐Closed Polyoxometalate Clusters {Mo180} and {Mo130Ce6}
Source: Angew Chem Int Ed Engl. 2017 Jun 1;56(33):9727–31. doi: 10.1002/anie.201702957 (PMC5600119; doi:10.1002/anie.201702957)
Supplement: Supplementary file 1 — Supplementary [file ANIE-56-9727-s001.pdf]

Angewandte Chemie
Eine Zeitschrift der Gesellschaft Deutscher Chemiker

Supporting Information

Exploring the Molecular Growth of Two Gigantic Half-Closed Polyoxometalate Clusters $\{Mo_{180}\}$ and $\{Mo_{130}Ce_6\}$

*Weimin Xuan, Robert Pow, De-Liang Long, and Leroy Cronin**

anie_201702957_sm_miscellaneous_information.pdf

Exploring the Molecular Growth of Two Gigantic Half-Closed Polyoxometalate Clusters $\{Mo_{180}\}$ and $\{Mo_{130}Ce_6\}$

Weimin Xuan, Robert Pow, De-Liang Long and Leroy Cronin*

WestCHEM, School of Chemistry, The University of Glasgow, University Avenue, Glasgow G12 8QQ, Scotland, UK; Fax: (+44)-141-330-4888;

E-mail: lee.cronin@glasgow.ac.uk;

Homepage: http://www.croninlab.com

Table of Contents

1. Materials S2
2. Instrumentation S2
3. Synthetic procedure of **1** and **2** S3
4. Structure analysis of **1** and **2** S4
5. Crystallographic data and crystal structures of **1-2** S9
6. References S13

1. Materials

Reagent-grade chemicals were obtained from Aldrich Chemical Company Ltd. and Alfa Aesar, and used without further purification.

2. Instrumentation

Crystallography: Suitable single crystal was selected and mounted onto a rubber loop using Fomblin oil. X-ray diffraction intensity data a Bruker Apex 2 CCD diffractometer (λ (MoK$_{\alpha}$) = 0.7107 Å) equipped with a microfocus x-ray source (50kV, 30w). Data collection and reduction were performed using the Apex2 software package and structure solution, and refinement were carried out using SHELXS-97[1] and SHELXL-97[2] using WinGX.[3] Corrections for incident and diffracted beam absorption effects were applied using empirical absorption correction. All the Mo atoms (including those disordered) and most of the O atoms were refined anisotropically. Solvent water molecule sites with partial occupancy were found and included in the structure refinement. Crystallographic formulas typically contain much more water molecules in the crystal lattice than the formulas used for chemical analyses as the sample was dried up. It is important to note that with these structures we are moving outside the realm of small molecule crystallography and are dealing with refinements and problems that lie between small molecule and protein crystallography. As a result we cannot expect refinements and statistics to follow the path of crystals with much smaller unit cells. However, the final refinement statistics are relatively good, and in all cases the structural analysis allows us to unambiguously fully determine the structures of the compounds. The X-ray crystallographic data reported in this article have been deposited at the Crystallographic Data Centres. For compound **1**, the data can be obtained free of charge from the Cambridge Crystallographic Data Centre via www.ccdc.cam.ac.uk/data_request/cif under deposition number CCDC-1536779. For compound **2** the data can be obtained from FIZ Karlsruhe, 76344 Eggenstein-Leopoldshafen, Germany (fax: (+49)7247-808-666; e-mail: crysdata@fiz-karlsruhe.de), on quoting the deposition number CSD-432719.

Fourier-transform infrared (FT-IR) spectroscopy: The samples were prepared as a KBr pellet and the FT-IR spectrum was collected in transmission mode in the range of 600-4000 cm^{-1} using a JASCO FT-IR 4100 spectrometer. Wavenumbers are given in cm^{-1}. Intensities are denoted as w = weak, m = medium, s = strong, br = broad, sh = sharp.

Element Analyses: Element analyses for Mo, Ce, K and Na were performed on a Leeman inductivity-coupled plasma (ICP) spectrometer while C, N and H content were determined by the microanalysis

services within the Department of Chemistry, University of Glasgow using an EA 1110 CHNS, CE-440 Elemental Analyzer.

Thermogravimetric Analysis (TGA): Thermogravimetric analysis was performed on a TA Instruments Q 500 Thermogravimetric Analyzer under nitrogen flow at a typical heating rate of 10°C min^{-1}.

3. Synthetic procedure of 1 and 2

1: $Na_4(C_5H_{11}N_2O_2)_2[H_{18}Mo_{180}O_{536}(H_2O)_{78}(C_5H_{10}N_2O_2)_7]\cdot 250\ H_2O$, M.W.: 33247.34

L-ornithine (6.2 mg, 0.047 mmol) and an aqueous solution of 0.1 M $[N_2H_4]\cdot 2HCl$ (0.5 mL) were added to to a solution of $Na_2MoO_4\cdot 2H_2O$ (242 mg, 1 mmol) in water (45 mL). The mixture was then acidified with 1 M $HClO_4$ (4.5 mL) to pH ~1.4. After heating with medium stirring in a 100-mL Erlenmeyer flask (widenecked; covered with a watch glass) at 90 ° C for 2 h. The resulting clear deep-blue solution was then cooled to room temperature, filtered and kept in an open 100-mL Erlenmeyer flask for three weeks. The deep-blue block-like crystals were collected by filtration, washed with ice-cold H_2O, and dried under inert atmosphere over $CaCl_2$, yield: 71 mg (30.5 % based on Mo). Elemental analysis, calc.: C, 1.62 %; H, 2.39 %; N, 0.76 %; Na, 0.28 %; Mo, 52.46 %, found: C, 1.67 %; H, 1.39 %; N, 0.86 %; Na, 0.328 %; Mo, 53.1 %; IR (KBr pellet, 4000–600 cm^{-1}): 3403(s, br), 1618 (m), 971 (m; ν (Mo=O)), 908 (w), 866 (w), 803 (s), 635 (s), 558 (s) cm^{-1}

2: $Ce_{0.5}[Ce_6H_{16.5}Mo_{130}O_{396}(H_2O)_{84}]\cdot 180\ H_2O$, M.W.: 24491.36

A solution of $CeCl_3\cdot 6H_2O$ (6.0 g, 16.4 mmol) in H_2O (300 mL) was quickly added under stirring to an aqueous solution of K_2MoO_4 (5.9 g, 24.8 mmol) in H_2O (300 mL). The yellow precipitate was collected by filtration after 30 min, washed with ice-cold H2O, and dried at 120 °C for 5 h. [4] To the solution of this precipitate (0.2 g), in a mixture of H_2O (45 mL) and 1 M $HClO_4$ (5.0 mL), an aqueous solution of 0.1 M $[N_2H_4]\cdot 2HCl$ (1.2 mL) and oxalic acid (5 mg, 0.055 mmol) were added. The solution was heated with medium stirring in a 100 mL Erlenmeyer flask (wide-necked; covered with a watch glass) at 90 °C for 2 h. The resulting clear deep-blue solution was then cooled to room temperature, filtered and kept in an open 100-mL Erlenmeyer flask for one week. The deep-blue block-like crystals were collected by filtration, washed with ice-cold H_2O, and dried under inert atmosphere over $CaCl_2$, yield: 75 mg (37.6 % based on Mo). Elemental analysis, calc.: K, 0 %; Na, 0 %; Mo, 50.92 %; Ce, 3.21 %; found: K, 0.018 %; Na, 0.024 %; Mo, 53.0 %; Ce, 4.06 %. IR (KBr pellet, 4000–600 cm^{-1}): 3408 (s, br), 2922 (w), 2851 (w), 1725 (w), 1608

(s), 1491 (m), 1407 (m), 1342 (m), 1251 (w), 1127 (w), 1055 (m), 964 (m; *v* (Mo=O)), 789 (s), 633 (s), 555 (s).

4. Structural analysis of 1 and 2

Although the wheel-type molybdenum blue architectures are very complex, the general approach to the structural analysis and formula determination is well documented.[4] The structural analysis requires the following lines of evidence / information to allow the assignment of formula and the structural details coupled with Single-crystal X-ray diffraction:

(i) Redox titration to help determine the number of reduced Mo^V centres (Uv-vis-NIR spectroscopy also can help corroborate this data via the analysis of the extinction coefficient for the LMCT associated with the reduced Mo^V centres. Each centre should contribute ca. 5 - 6 x 10^3 L $mol^{-1}\cdot cm^{-1}$ to ε).

(ii) Bond valence sum analysis to confirm the terminal oxo positions, reduced Mo^V centres and the positions of the hydroxide ligands.[5]

(iii) Elemental analysis of sodium, molybdenum, cerium and C, H, N analysis.

(iv) TGA to estimate the number of ligand and solvent water molecules.

Therefore, the analysis below both presents this data and demonstrates how the structural assignment is consistent with this data.

Redox titrations

The cerimetric titration was carried out using a 0.005 M solution of Ce^{IV} in 0.5 M of sulphuric acid as oxidant which was added dropwise to a solution of compound **1** (20 mg in 50 mL of H_2O). After addition of 3.93 mL of the oxidant the colour of the solution turned from deep blue to colourless along with a characteristic potential jump showed the presence of 32 ± 1 4d electrons which (formally) corresponds to 32 Mo^V centres (theoretical value for 32 e- reduced species : 3.84 mL) . The cerimetric titration of **2** was performed in the same way as **1**, and 4.00 ml of 0.005 M solution of Ce^{IV} was used, which is consistent with theoretical value for 24 e- reduced species (3.92 mL).

Bond valence sum analysis

Table S1. Average Bond valence sum values for the Mo centres which span the incomplete $\{Mo_5O_6\}$-type double cubanes and the μ_3-O atoms of the $\{(\mu_3\text{-}O)_2O_2\}$-type compartments in **1** and **2**.

Compounds	BVS (Mo)	BVS (μ_3-O)
**1**	5.59	1.23
**2**	5.58	1.27

Elemental analysis and C, H, N analysis

See Section 3. Synthetic procedure of **1** and **2**

Uv-vis-NIR spectra and TGA curves

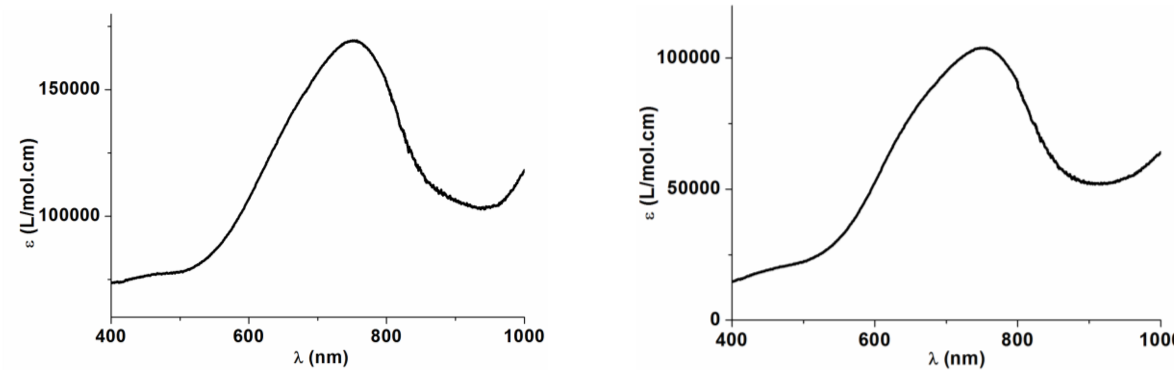

Figure S1 Uv-vis-NIR spectra of **1** (left) and **2** (right) in water (2×10^{-6} mol/L). The average ε of each Mo^{V} centre is about 5.30 x 10^3 and 5.20 x 10^3 L $mol^{-1}\cdot cm^{-1}$ for **1** and **2** at 753 nm corresponding to the LMCT, respectively.

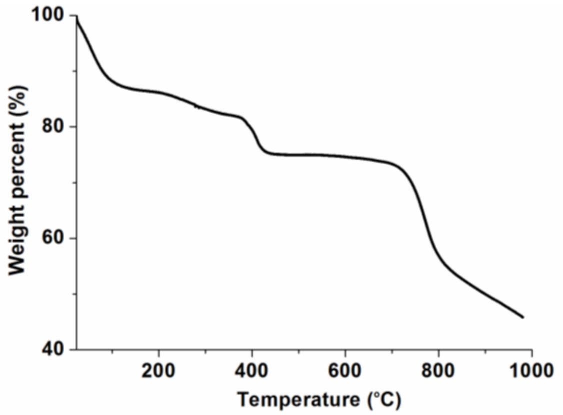

Figure S2 TGA curve for compound **1**.13.50% weight loss corresponds to ~250 H_2O.

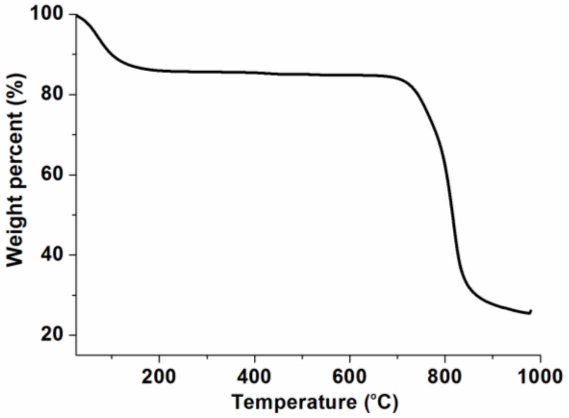

Figure S3 TGA curve for compound **2**.13.2% weight loss corresponds to ~180 H_2O.

Summary of structure analysis

Compound 1 : Firstly, the overall reduction state of **1** (32 electrons reduced) was confirmed using three independent techniques (Uv-vis-NIR spectroscopy, redox titration and bond valence sum analysis). Because of the presence of {Mo_{30}} cap on top of {Mo_{150}}, more sites are available for reduction compared with archetypal {Mo_{154}} (28 electrons reduced) and thus result in 32 electrons-reduced **1**. The further characteristic of the structure of the anion **1** is the large number of protons resulting from the 32 e^- reduction. A careful analysis of the bond length of Mo-O bonds and bond valence sums reveals 18 singly and 78 doubly protonated oxygen atoms (coordination water). Singly protonated are the 14 equivalent μ_3-O atoms situated in the incomplete double-cubane-type {Mo_5O_6} and several terminal O atoms that have bond lengths of Mo-O in the range of 2.0 to 2.2 Å. The terminal O atoms that form bond longer than 2.2 Å are assigned as coordination water. All the bridging O atoms are considered as O^{2-} except for the 14 equivalent μ_3-O atoms. In this way, we could determine the overall charge of **1a** in **1** as -6. Elemental analysis result of Mo confirms the framework of **1** consists of 180 Mo atoms, consistent with single-crystal x-ray diffraction. To balance the negative charge of -6, four sodium ions and two protonated L-orinithine are proposed as counterions based on elemental analysis result of Na. The amount of L-ornithine is deducated from C, H, N analysis and there are in total nine L-ornithine in the structure of **1**. Among them, seven are located on the framework of **1a** while another two are guest molecules. Finally, TGA curve of **1** exhibits a total weight loss of 13.5 % from r.t. to 150 °C, which corresponds to ~250 guest water molecules.

The archetypal ring {Mo_{154}} contains consists of 14 sets of three different building block types: {Mo_8}, {Mo_1} and {Mo_2}. In a similar way, we can determine the composition of {Mo_{150}} in **1** as $[\{Mo_2\}_{12}\{Mo_1\}_{14}\{Mo_8\}_{14}]^{18-}$ by deleting two {Mo_2} unit from {Mo_{154}}. The formula of {Mo_{30}} cap could be evaluated as $[\{Mo_2*\}_2\{Mo_2^{®}\}\{Mo_8^{©}\}_3]^{12+}$ based on the aggregation of three {Mo_8} units, two edge-

shared $\{Mo_2*\}$ units and one corner-shared $\{Mo_2^{®}\}$unit. Taking into consideration of the obtained information from the above calculations along with Single-crystal x-ray diffraction, elemental analyses, bond valence sum analysis, Uv-vis-NIR, TGA and redox titration, it is possible to determine the overall building-block scheme and overall formula for **1** as $Na_4(C_5H_{11}N_2O_2)_2[\{Mo_2\}_{12}\{Mo_1\}_{14}\{Mo_8\}_{14}\{Mo_2*\}_2\{Mo_2^{®}\}\{Mo_8^{©}\}_3] \equiv Na_4(C_5H_{11}N_2O_2)_2[\{Mo^{VI}_2O_5(H_2O)_2\}_5\{Mo^{VI}_2O_5\}_7\{Mo^{VI/V}_8O_{26}(\mu_3O)_2H(H_2O)_3Mo^{VI/V}\}_{14}(C_5H_{10}N_2O_2)_7\{Mo^{VI}_2O_5(H_2O)*\}_2\{Mo^{VI}_2O_4(OH)_4^{®}\}\{Mo^{VI/V}_8O_{22}(H_2O)_8\}_3^{©}]\cdot ca.250H_2O$

Compound 2 : The formula of **2** is determined in a similar way to **1**. Firstly, the overall reduction state of **2** (24 electrons reduced) was confirmed using three independent techniques (Uv-vis-NIR spectroscopy, redox titration and bond valence sum analysis). A careful analysis of the bond length of Mo-O bonds and bond valence sums reveals 12 singly and 84 doubly protonated oxygen atoms (coordination water). Accordingly, the overall charge of **2a** in **2** is -2. Elemental analysis indicated the presence of 130 Mo and 6 Ce on the framework of **2a** as well as 0.5 Ce ion as counterion. TGA curve of **2** exhibits a total weight loss of 13.2% from r.t. to 150 °C, which corresponds to ~180 guest water molecules.

The parent $\{Mo_{120}Ce_6\}$is composed of 12 $\{Mo_8\}$ units, 6 $\{Mo_2\}$ units, 12 $\{Mo_1\}$ units, and 6 $\{Ce(H_2O)_5\}$ units (Note that: one $\{Ce(H_2O)_5\}$ replaces one$\{Mo_2\}$ unit on the rim of wheel). Because $\{Mo_{120}Ce_6\}$ has the exactly same composition of reported $\{Mo_{120}Pr_6\}$ except for lanthanide ions, so we just adopt the formula directly from $\{Mo_{120}Pr_6\}$. For more detailed information about how to calculate the formula of lanthanide-doped Mo Blue, please refer to published procedures. The formula of $\{Mo_{10}\}$ cap could be evaluated based on the aggregation of one pentagonal $\{Mo_6\}$ unit and two corner-shared $\{Mo_2\}$ units. In summary, the overall building-block scheme and overall formula for **2** is determined as $Ce_{0.5}[\{Mo_2\}_6\{Mo_1\}_{12}\{Mo_8\}_{12}\{Mo_{10}\}\{Ce\}_5] \equiv Ce_{0.5}[\{Mo^{VI}_2O_5(H_2O)_2\}_6\{Mo^{VI/V}_8O_{26}(\mu_3\text{-}O)_2H(H_2O)_3Mo^{VI/V}\}_{12}\{Mo^{VI}_{10}O_{26}(OH)_4(H_2O)_6\}_2\{Ce(H_2O)_5\}_6H_{0.5}]\cdot ca.\ 180H_2O$

5. Crystallographic data and crystal structures of 1-2

Table S2. Crystal data and structure refinement for **1**

Identification code	**1**
Empirical formula	C45 H766 Mo180 N18 Na4 O882
Formula weight	33037.88
Temperature (K)	150(2)
Wavelength (Å)	0.71073
Crystal system	Triclinic
Space group	*P*-1
Unit cell dimensions	a = 33.0710(14) , α = 81.765(2)º<br>b = 37.4645(16) , β = 71.111(2) º<br>c = 42.3985(17) , γ = 83.367(2)º
Volume (Å^3), Z	49056(4) , 2
Density (calculated) (mg/m^3)	2.237
Absorption coefficient (mm^{-1})	2.326
F(000)	19586
Crystal size (mm^3)	0.100 x 0.070 x 0.070
θ range for data collection (º)	0.692 to 26.000
Limiting indices	40<=h<=40, -46<=k<=46, -52<=l<=52
Reflections collected	725102
Independent reflections	192434 [R(int) = 0.0551]
Completeness to theta	25.242/ 99.8 %
Absorption correction	Empirical
Max. and min. transmission	0.754 and 0.531
Refinement method	Full-matrix least-squares on F^2
Data / restraints / parameters	192434 / 46 / 8866
Goodness-of-fit on F^2	1.191
Final R indices [I>2sigma(I)]	R1 = 0.1077, wR2 = 0.2671
R indices (all data)	R1 = 0.1708, wR2 = 0.3592
Largest diff. peak and hole (e.Å^{-3})	2.99 and -3.66

Table S3. Crystal data and structure refinement for **2**

Identification code	**2**
Empirical formula	Ce6.50 H544.50 Mo130 O660
Formula weight	24491.80
Temperature (K)	150(2)
Wavelength (Å)	0.71073
Crystal system	Orthorhombic
Space group	*P*nma
Unit cell dimensions	a = 31.306(6), α = 90 º<br>b = 42.954(8), β = 90 º<br>c = 48.315(9), γ = 90 º
Volume ($Å^3$), Z	64970(21), 4
Density (calculated) (mg/m^3)	2.504
Absorption coefficient (mm^{-1})	2.979
F(000)	46646
Crystal size (mm^3)	0.100 x 0.080 x 0.050
θ range for data collection (º)	0.775 to 26.00
Limiting indices	-37<=h<=38, -52<=k<=52, -58<=l<=59
Reflections collected	432108
Independent reflections	64712 [R(int) = 0.0692]
Completeness to theta	25.242/ 99.9 %
Absorption correction	Empirical
Max. and min. transmission	0.745 and 0.467
Refinement method	Full-matrix least-squares on F^2
Data / restraints / parameters	64712 / 0 / 3606
Goodness-of-fit on F^2	1.233
Final R indices [I>2sigma(I)]	R1 = 0.0850, wR2 = 0.1714
R indices (all data)	R1 = 0.1450, wR2 = 0.2356
Largest diff. peak and hole (e.$Å^{-3}$)	3.83 and -2.41

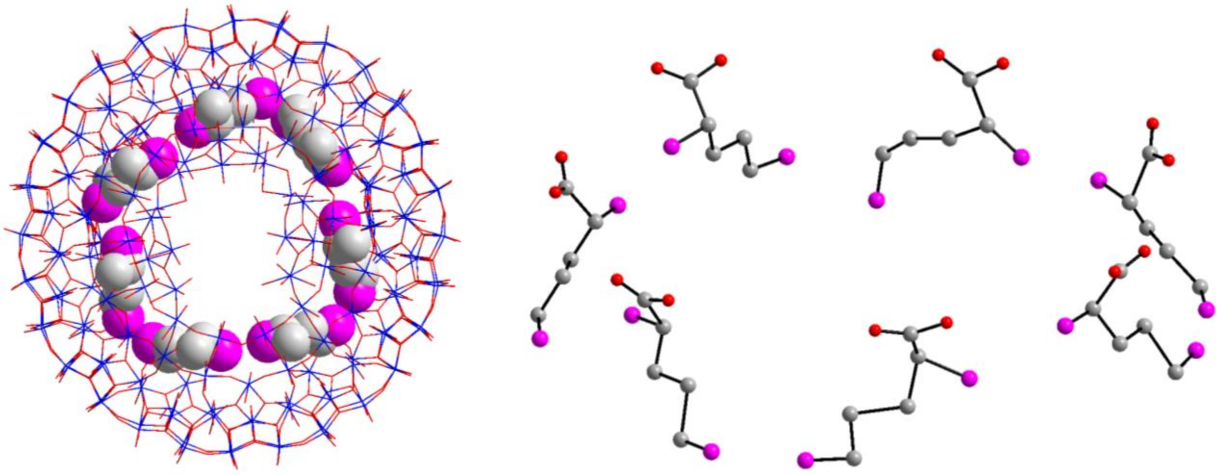

Figure S4. LEFT: View of the arrangment of L-ornithine in space filling mode within **1a**. RIGHT: View of head-to-head and tail-to-tail orrientation of amino groups on adjacent L-ornithine in ball and stick mode. Mo, blue; O, red; C, gray; N, pink.

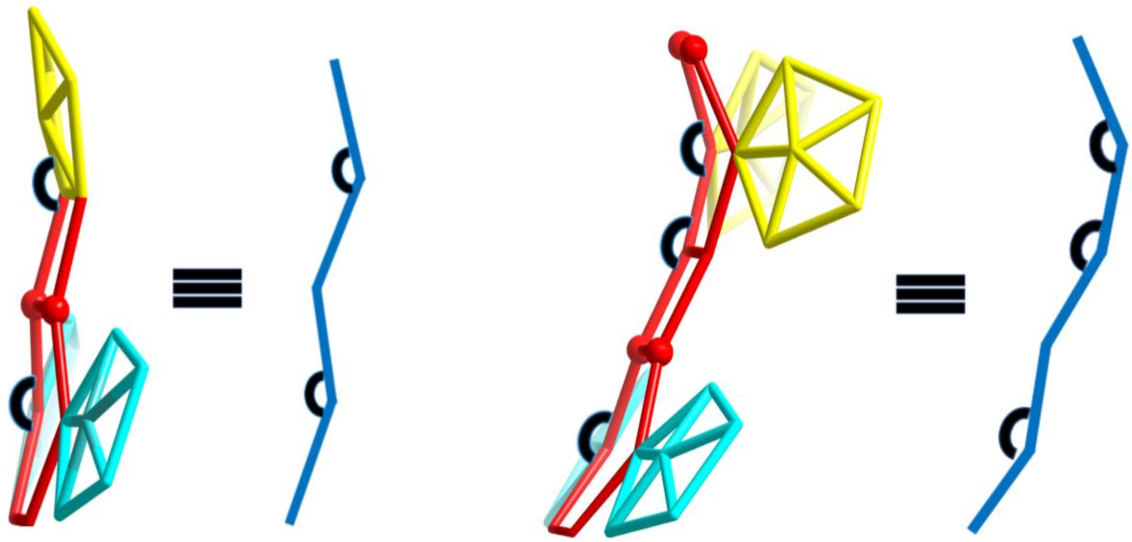

Figure S5. View of the two domains of negative curvatures between layer B and layer C in **1a**. Pentagonal $\{Mo_6\}$ are represented in cyan for layer B and yellow for layer C. $\{Mo_2\}$ units are highlighted in red balls. Black arcs are used to indicate the negative curvatures. The blue lines show schematic representation of the negative curvatures.

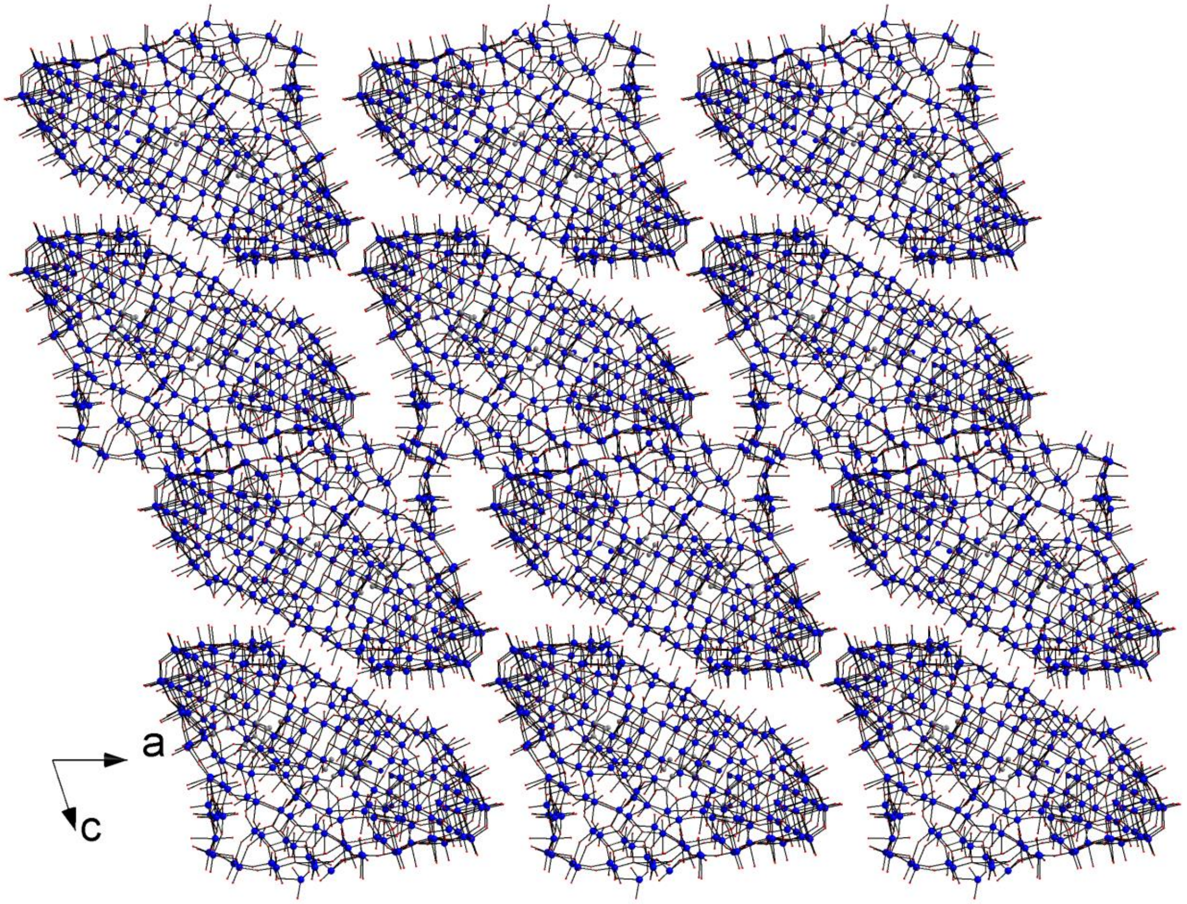

Figure S6. Rpresentation of packing diagram of **1a** along *b* axis. Mo, blue; O, red; C, gray; N, pink.

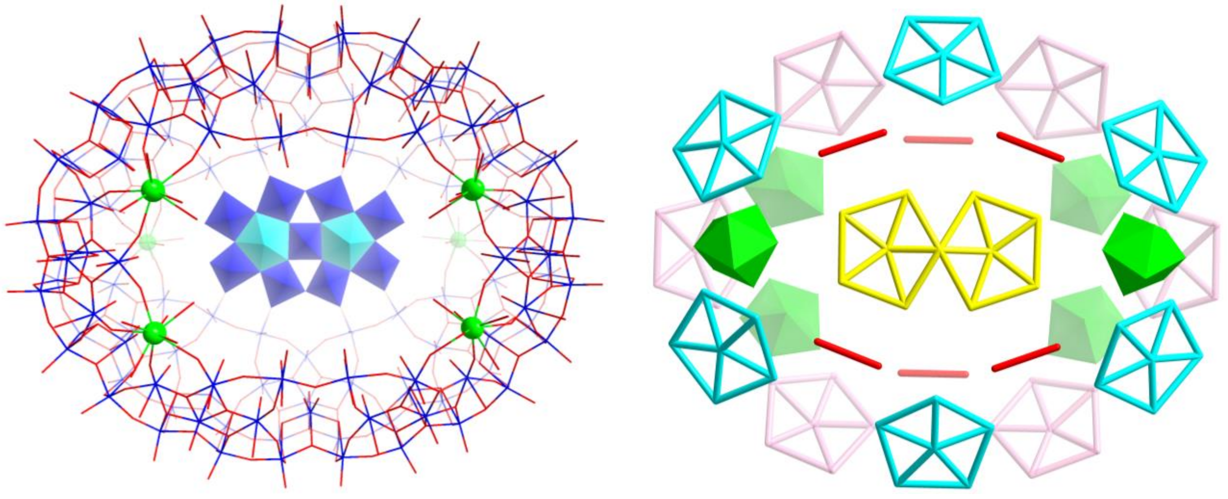

Figure S7. LEFT: View of the molecular structure of **2a** in wires mode with the ideal {Mo_{11}} fused pentagon in polyhedron and Ce ions in green ball. Mo, blue; O, red**,** Ce, green; {Mo_{11}}, blue polyhedra with central pentagonal Mo in cyan. RIGHT: Simplified representaion of **2a** based {Mo_6} pentagons, {Mo_2} units and Ce ions. {Mo_{11}}, yellow sticks; {Mo_6}, cyan for upper part and rose for lower part; {Mo_2}, red sticks; Ce, green polyhedra.

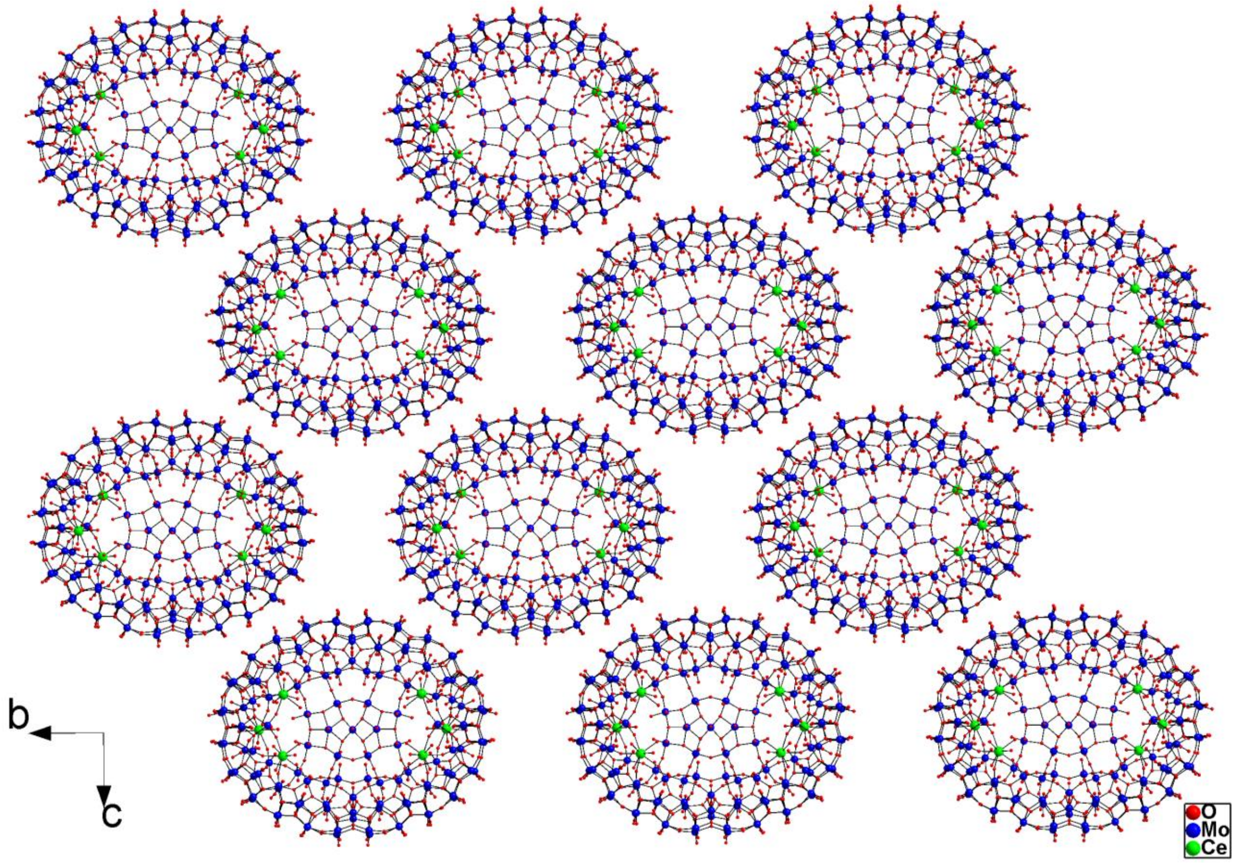

Figure S8. Rpresentation of packing diagram of **2a** along *a* axis. Mo, blue; O, red; Ce, green.

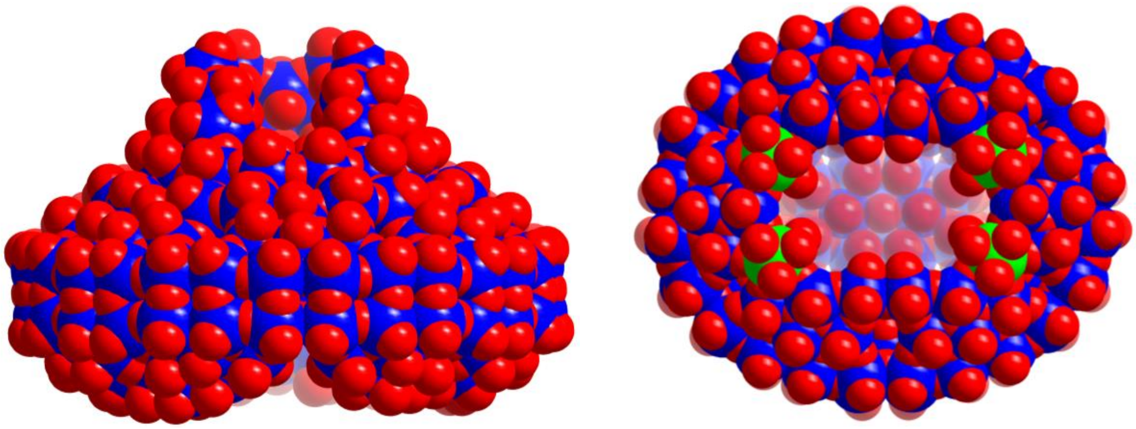

Figure S9. Space filling model of **1a** (left) and **2a** (right). Mo, blue; O, red; Ce, green.

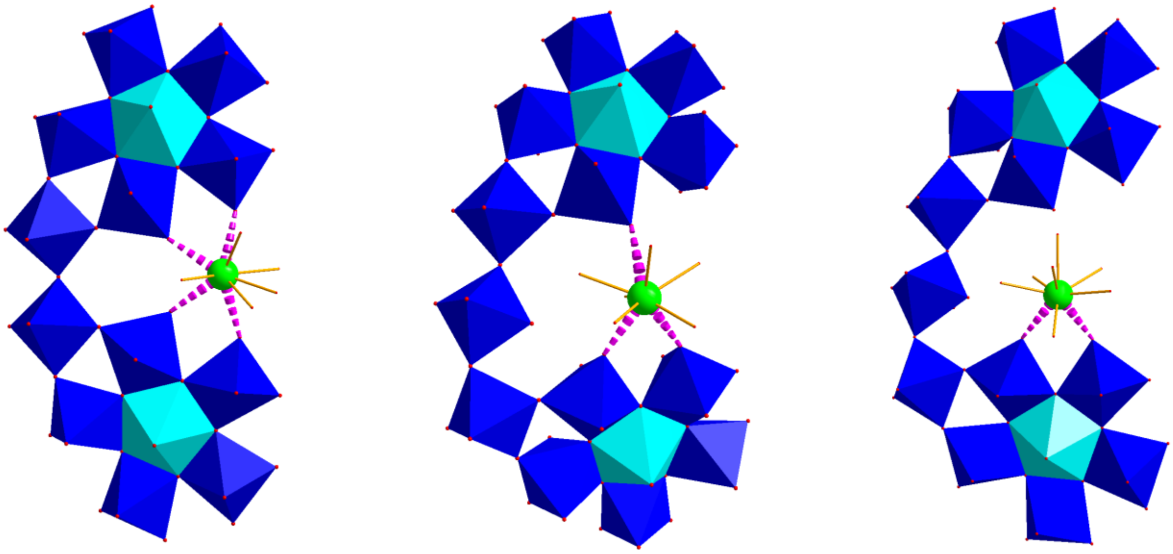

Figure S10. Rpresentation of the three different connecting modes of Ln^{III} ions with adjacent $\{Mo_6\}$ pentagons in LMB. LEFT: 4-connected mode; MIDDLE: 3-connected mode; RIGHT: 2-connected mode. $\{Mo_1\}$, yellow polyhedron; $\{Mo_2\}$, red polyhedron; $\{Mo_8\}$, blue polyhedron with central pentagonal unit in cyan polyhedron; Ln^{III}, green ball; O, red ball. The connection between Ln^{III} and $\{Mo_6\}$ pentagons is highlighted by dotted purple bonds.

6. Reference

1. G. Sheldrick, *ActaCrystallographica Section A*, **1990**, *46*, 467-473.
2. G. Sheldrick, *ActaCrystallographica Section A*, **2008**, *64*, 112-122.
3. L. Farrugia, *J. Appl. Crystallogr.*, **1999**, *32*, 837-838.
4. a) A. Müller, E. Krickemeyer, H. Bögge, M. Schmidtmann, C. Beugholt, S. K. Das, F. Peters, *Chem. Eur. J.* **1999**, 5, 1496-1502; b) A. Müller, C. Serain, *Acc. Chem. Res.* **2000,** *33*, 2-10.
5. I. D. Brown, In Structure and Bonding in Crystals, Vol. II (Ed.: M.O‘Keefe, A. Navrotdsky, Academic Press, New York, 1981), p. 1.
